# Do working characteristics influence the participation at health measures? Findings from a trial phase of workplace health promotion

**DOI:** 10.1186/s12995-020-00262-3

**Published:** 2020-05-19

**Authors:** Annika Reinhardt, Johanna Adams, Klaus Schöne, Dirk-Matthias Rose, Stefan Sammito

**Affiliations:** 1 Institute of Teachers’ Health at the University Medical Center of the Johannes Gutenberg University of Mainz, Kupferbergterrasse 17-19, 55116 Mainz, Germany; 2 Bundeswehr Medical Service Headquarters, Section Health Promotion Sport and Nutrition Medicine, Koblenz, Germany; 3 Research & Development, Air Force Centre of Aerospace Medicine, Cologne, Germany

**Keywords:** Occupational health management, Military, Health promotion, Health settings, Germany

## Abstract

**Background:**

Health behavior is presumed to be influenced by organizational factors. This study analyzes how workplace characteristics influence health behavior in terms of participation at health measures.

**Methods:**

Employees of the German Federal Ministry of Defense were surveyed at the beginning (January / February 2015) and at the end (June 2015) of the trial phase of workplace health promotion (WHP). Differences in participation of characteristic groups were calculated using Pearson’s Chi^2^-Test and T-Test, chances of participation were estimated using multilevel logistic regression.

**Results:**

Employees who reported higher satisfaction with work demand participated more often in health measures (aOR: 1.02, 95%-CI = 1.01, 1.04, *p* < 0.001). Large amount of variance in participation can be attributed to department level.

**Conclusion:**

Participation at WHP varies significantly between settings after controlling for individuals’ characteristics. Thus, working characteristics should be considered as a decisive factor for WHP effectiveness. There is consensus that behavioral prevention is most effective when conditional prevention is granted as behavior is presumed to be influenced by individuals´ environmental conditions. Though objective working conditions may seem similar further context characteristics which remain unconsidered may lead to different behavior patterns. This article shows that more attention must be payed to setting specific characteristics with regard to effective Occupational Health Promotion.

This project is registered by the Federal Ministry of Defense (research number: E/U2AD/ED003/EF555).

## Background

Since the Ottawa Charter for Health Promotion stresses the importance of settings for individual health and well-being [1], it has become clear that health promotion has to focus on a broad set of factors influencing health behavior to be successful. A setting can be defined as a social and physical context in which people are frequently engaged and interact with their environment [2, 3]. The workplace is one of the most important settings with regard to the large amount of time people spend working and the various kind of work-associated strains and health implications [4]. In terms of workplace health promotion (WHP) besides socio-demographic characteristics and attitudes of the employees, physical, social and organizational aspects of the workplace must be taken into account.

There is consensus that behavioral prevention is most effective when conditional prevention, that is to say that working conditions are designed to be as healthy as possible, is granted [3–8]. The objective of this study was to investigate how the perception of employees of their workplace setting influences their interest at WHP and the associated health measures. The study’s hypothesis was that employees who assess their working characteristics overall positive are more likely to participate at health measures. Health measures include all programs and courses for health promotion offered by the employer. Since the individual assessment of working characteristics is affected by general conditions of the workplace it was further expected that perceived working characteristics and thus participation in health measures differ between organizations. Thus, it seemed necessary to control for context effects which were expected to moderate the relationship between individual’s assessment and the likelihood of participation.

A key indicator for the effectiveness of WHP is the participation rate. For workplace intervention the reach is usually below 50% [9, 10] and influenced by individual as well as business characteristics. For example, previous research revealed an overall higher participation rate for women [10–14], higher educated staff [10], employees with high job satisfaction [15] and an increase in participation and compliance with increasing age [12, 15–17]. Concerning the health status no consistent relation is reported. Whereas some studies showed evidence that people with a good health status participate more often in health measures others refer the opposite [9, 16, 18, 19]. However, persons who generally show a health-oriented behavior are more likely to attend [11, 12, 20]. On the macro level participation is influenced by the number of employees in a company whereby a decrease in participation rates with increasing personnel can be observed [15, 21]. Also aspects of the organizational structure like flexible working hours [13, 19, 22] or the social climate respectively the extent of social support [4] within an organization already proved to affect individual’s health behavior and thus participation at health measures.

The aim of this study is to examine to what extent context effects also affect the health behavior and the interest in health measures on the part of the employees in the MoD division. The authors hope to gain deeper insight into factors that can influence the success of WHP.

## Methods

This work was supported by German Federal Ministry of Defense (MoD) and approved by the ethics commission of the State Chamber of Physicians of Rhineland-Palatinate. The presented data was collected in two waves as part of the trial phase of WHP in the MoD. The WHP was fully funded by the employer, the MoD, with the long-term goal of implementing Occupational Health Management in the entire business area. During the trial phase, employees at eleven departments were able to take part in the health measures offered on site during their working hours. The measures were offered from February 2015 over the entire study period. The first survey wave was conducted at the beginning of the trial phase in January / February 2015 and the second survey wave proceeded in June 2015 at the end of the trial phase. All employees at the eleven pilot departments were able to take part in the survey voluntarily. The first questionnaire covered working characteristics, well-being, health behavior and lifestyle whereas the second questionnaire included questions concerning employer attractiveness and commitment, based on the agreement of values and the feeling that the employer takes appropriate care of the health of its employees, as well as attitudes towards WHP and participation at four kinds of health measures: (a) “physical activity”: Measures aiming at increasing physical activity of employees by offering certain sports courses; (b) “addiction”: information events to prevent addiction of legal drugs; (c) “nutrition”: mainly information provided by nutritionist; (d) “stress and sleep”: courses and coaching in order to teach relaxation techniques, improve sleep quality and enhance mental balance. Not all health measures were offered in every department and physical activity courses account for the vast majority of all measures in each department. Eleven departments which are considered to be representative for the personnel structure of the MoD were selected as units in the trial phase including operational forces, military hospital and administrative bodies.^1^ Overall 9297 employees could have participated either in a written or an online survey. Because the focus of interest is the relation between working characteristics and participation at health measures, analyses were feasible for participants at both surveys only.

In a first step, bivariate relations between participation behavior and socio-demographic variables among the four kinds of health measures were analyzed using Pearson’s chi-squared test. Further, differences in means of the assessment of working characteristics between participants and non-participants were tested using independent t-test.

As all 33 items which have been applied to assess working characteristics could be answered on a four-staged scale (1“no”; 2”rather no”; 3”rather yes”; 4“yes) and are therefore not interval-scaled, it was necessary to recode them into binary variables for statistical analysis. The individual assessment of work characteristics on the basis of those items can theoretically be divided in four dimensions: work demand, work amount, social climate, and environmental aspects. Confirmatory factor analysis was used to verify presumed dimensionality and confirmed acceptable internal consistency^2^ for work amount (6 Items; α = 0.77), work demand (11 Items; α = 0.70), and social climate (12 Items; α = 0.82) but marginally acceptable internal consistency for environmental aspects (4 Items; α = 0.60). However, the environmental aspects scale only includes four items and thus reliability can be regarded as sufficient [24].

In a third step, the effects of individual as well as department characteristics on participation in health measures were assessed in order to get more detailed insight how these variables influence the likelihood of participation under control of the other explanatory variables. WHP participation was operationalized as dichotomous variable, where 0 represents no participation at all and 1 represents participation in one of the measures offered for at least once. With respect to the hierarchical data structure we used multilevel logistic regression to estimate the effects on participation at health measures.

Regarding previous findings women and elders were expected to show higher participation. Furthermore, occupational status and perceived working characteristics were presumed to influence WHP participation, whereby (highly) satisfying working characteristics are expected to be related with higher WHP participation. Military personnel are obliged to a certain extend of physical activity within their employment relationship and thus can be expected to have lower interest particularly in sport programs.

On department level, an increasing number of employees was expected to be associated with reduced participation. Because sex and occupational status were expected to influence participation behavior on individual level, we also tested for comparable aggregated effects of gender ratios and occupational group percentages for each department by controlling for influences of level-1 predictors.

All statistical analysis were performed using STATA 14.0.

## Results

The response rate in the first survey (22.3%, *n* = 2076) was higher than in the second survey (15.9%, *n* = 1481). 24.2% of the first-wave-participants repeated participation in the second wave (*n* = 502). Sociodemographic distribution of all samples is reported in Table 1.

**Table 1:** Sociodemographic distribution of participants (%) in both survey waves

	Participants in February/March 2015	Participants in both surveys	Participants in June 2015
**sex**			
male	62.96	71.12	66.91
*n*	*991*	*357*	*655*
female	28.02	28.69	23.70
*n*	*441*	*144*	*232*
missing	9.02	0.20	9.40
*n*	*142*	*1*	*92*
**age**			
Below 30	30.24	30.88	30.54
*n*	*476*	*2*	299
30 - 39	21.22	21.31	21.14
*n*	*334*	*107*	207
40 - 49	20.71	20.52	20.53
*n*	*326*	*103*	201
older than 49	19.44	21.51	19.61
*n*	*306*	*108*	192
missing	8.39	5.78	8.17
*n*	*132*	*29*	80
**occupational status**			
civil servants	35.58	36.25	30.64
*n*	*560*	*182*	*300*
military staff	53.88	63.15	61.29
*n*	*848*	*317*	*600*
missing	10.55	0.60	8.07
*n*	*166*	*3*	*79*
response rate	16.93	5.40	10.53
**n**	**1574**	**502**	**979**

The overall reported participation rate at health measures of 61.6% is comparatively high for workplace interventions but varies considerably between the four kinds of measures offered. Hence there is a relatively high participation at physical activity measures of almost 50%, and almost a third of employees survey participated in nutrition measures but participation rates for measures referring to addiction or stress and sleep are quiet low (Table 2). With respect to individual characteristics, significant differences in participation can be reported for health measures concerning stress and sleep, physical activity and for overall participation. Compared to the gender distribution in the overall sample, men rarely participate in health measures disproportionately. Women participated more often in all measures, except addiction. Overall, 74% of the women surveyed, but only 57% of the men surveyed, participated in one or more measures. The gender difference is significant for participation at health measures as a whole and for physical activity in particular. Though there are also significant differences of participation between the age groups, no consistent pattern can be identified. Older persons tend to participate in physical activity measures more likely than younger age groups and employees older than 49 years show the highest overall participation rate. But this group also shows the lowest participation rate for measures on the subject of addiction and the second smallest participation rate for nutrition measures. The participation rate of the youngest age group varies considerably between the four kinds of measures but always remains beyond 50%. A significantly high demand for measures concerning stress and sleep compared to the other age groups can be reported for employees between 40 to 49 years. But it cannot be derived that certain age groups have certain patterns of interests or needs.

**Table 2:** Differences in participation rates (%) of characteristic groups: results from Pearson’s Chi^2^-Test

	Physical Activity (***n*** = 502)	Addiction (***n*** = 474^**a**^)	Nutrition (***n*** = 396^**a**^)	Stress and Sleep (***n*** = 502)	Overall measures (***n*** = 502)
**Overall participation**	47.6	10.8	28.9	19.1	61.6
**Sex**					
Male	42.7**	17.5	41.5	20.3	56.6**
Female	69.1**	12.5	44.3	25.4	73.6**
**Age**					
Below 30	22.8**	12.8	38.0	11.8*	45.2**
30-39	50.0	20.9	43.5	18.6	65.5
40-49	61.2*	23.8	47.8	30.1*	68.0
older than 49	74.8**	9.4	38.9	26.4	75.9**
**Occupational status**					
Civil servants	73.0**	17.8	44.8	28.9*	76.4**
Military staff	36.9**	15.8	40.4	17.7*	53.0**

Boldface indicates statistical significance (*p < 0.05, **p < 0.001)

^a^ Sample size of *n* = 502 employees over all departments is reduced because this kind of health measure was not offered in all departments (9 out of 11 for both kinds)

Military servants are significantly underrepresented in the scope of physical activity whereas three out of four civil servants reported participation at physical activity measures. However, the military staff’s participation is lower in each kind of measure compared to civilians and thus – though more than half of all military servants surveyed took part in at least one of the measures – the participation rate of civil servants is overall significantly higher.

As can be derived from Table 3 there are partially significant differences in the assessment of work characteristics between participants and non-participants. All dimensions have been rated higher by participants compared to non-participants. Especially the group of participants in physical activity measures evaluated every aspect of work characteristics more positive compared to non-participants at a significant level. Work amount ratings differ significantly for all kinds of measures - except stress and sleep - whereas differences in the rating of work demand occurs solely at an insignificant level for addiction measures.

**Table 3:** Differences in means of rating scales for perceived working characteristics between participants and non-participants

		Work Amount	Work Demand	Social Aspects	Environ-mental Aspects
**Physical Activity**	Participants (*n* = 239)	0.72*	0.77**	0.84**	0.81*
	Non-Participants (*n* = 235)	0.64*	0.67**	0.76**	0.75*
**Addiction**	Participants (*n* = 54)	0.76*	0.76	0.83	0.83
	Non-Participants (*n* = 271)	0.66*	0.71	0.80	0.78
**Nutrition**	Participants (*n* = 145)	0.73*	0.75*	0.82	0.78
	Non-Participants (*n* = 200)	0.66*	0.70*	0.81	0.79
**Stress and Sleep**	Participants (*n* = 96)	0.70	0.75*	0.79	0.81
	Non-Participants (*n* = 349)	0.67	0.70*	0.79	0.76
**Overall**	Participants (*n* = 309)	0.71**	0.76**	0.82*	0.79*
	Non-Participants (*n* = 193)	0.61**	0.65**	0.75*	0.75*

Means of scales were calculated by sum of ratings (0 for disagreement / dissatisfaction and 1 for agreement / satisfaction) divided by total number of answers per scale

Boldface indicates statistical significance (*p < 0.05, **p < 0.001)

In order to get more detailed insight beyond the distribution of single characteristics between participants and non-participants and to examine how specific characteristics influence overall participation when other explanatory variables are controlled the results of hierarchical logistic regression model are presented in Table 4. Among the level-1-variables age and sex prove to be substantial determinants of participation over all models. Men show a significantly lower chance for participation than women and the chance for employees of participation increases with increasing age. No differences could be found between military and civil servants. The general perceived health situation proves to have insignificant relevance for participation.

**Table 4:** Participation at organizational health measures: Odds ratios from hierarchical logistic regression model

	Model 1	Model 2	Model 3	Model 4
*Level 1 intercept*	2.78	1.61	7.78	18.67
Sex [ref.: female]	0.40**	0.30**	0.29**	0.28**
Health status	1.17	1.04	1.05	1.05
Age [ref.: < 29]				
30 – 39	2.02*	2.15*	2.24*	2.22*
40 – 49	2.29*	2.55*	2.58*	2.52*
50+	3.05**	3.10*	3.18*	3.08*
Occupational status	0.67	0.98	0.93	0.96
Social Aspects		0.99	0.99	0.99
Work amount		1.01	1.01	1.01
Work demand		1.02**	1.02**	1.02**
Environmental Aspects		0.99	0.99	0.99
*Level 2 Variables*				
Unit size			0.99*	0.99
Share of female employees				0.97
Share of military servants				0.99
Unit level variance	1.29**	1.27**	0.91**	0.88**
Observations	465	401	401	401
Log likelihood	− 258.6050 ^a^	− 221.3645 ^a b^	−219.3125 ^a b^	−218.9026 ^a^
ICC	0.34	0.33	0.20	0.19

Boldface indicates statistical significance (*p < 0.05, **p < 0.001)

^a^ Model proved better goodness of fit compared to the non-hierarchical logistic model according to LR-Test

^b^ Model proved better goodness of fit compared to the previous model according to LR-Test

No substantial effects on participation can be reported among the working characteristics social climate, quantity of work, or environmental aspects. Only work demand shows a significant effect indicating that the more positive the evaluation of work demand is, the more increases the chance of attendance at health measures.

**Fig. 1:**
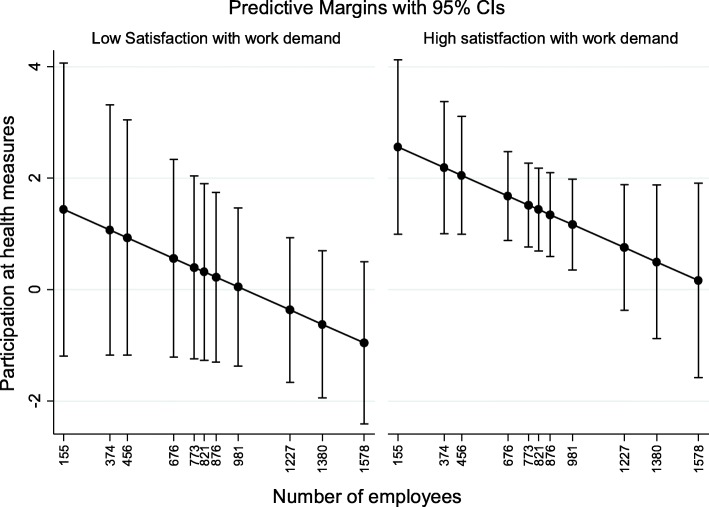
Participation rate at WHP depending on the departement's number of employees and individual's satisfaction with work demand

In every model the context specific intercept varies significantly suggesting that the influence of the independent level-1-variables varies between the eleven departments. However, between-context-variance decreases considerably when level-2-variables are added to the model. This is particularly the case if the analysis is controlled for the number of employees within a department (Model 3). The participation in dependence of the number of employees separated according to high and low satisfaction with work demand is depicted in Fig. 1. The likelihood of participation in at least one of the health measures decreases as the number of employees within a department increases. This relationship is the same for both employees with high and with low satisfaction concerning their work demand. But it is also apparent that employees with higher satisfaction show a higher chance of participation in each department. Though these differences could lead to the assumption that the negative effect of an increasing number of employees can be partially mitigated by high satisfaction with work demand no (significant) cross-level-interaction effect could be verified.

The intraclass correlation coefficient (ICC) is relatively high when only level-1-variables are included in the model. In the models 1 and 2 more than one third of variance is attributed to the department level. However, when controlling for the number of employees in model 3 the proportion of variance explained by the context as well as the level 2 intercept variance is clearly reduced. A larger proportion of variance of the dependent variable seems to be explained by differences in the number of employees. Regarding a comparably slight reduction of ICC in model 4 no substantial effect on the variance of participation between the departments can be reported for the context variables share of military servants or share of females. Furthermore, the between-context-variance in model 4 compared to model 3 is only little reduced and the log likelihood is also marginally decreased.

## Discussion

The present study refers on data in the sphere of responsibility of the MoD, a workplace with particular requirements and many threats to physical and mental health especially for the active military personnel [25, 26]. Though there is a pre-selection of people in a good health in military service [27–29], a constant demand for flexibility and high performance, both physically and mentally, the exposure to hazardous situations and the ensuing risk of traumatic experiences, missions far from home and being away from family jeopardize the health status of soldiers [4, 30, 31]. On the other hand the sample includes other occupational groups like civil servants or hospital workers who are quite comparable to general working population. Their work strain was expected to be similar to other administration or health care staff. Nevertheless, this sample is not fully comparable to “regular” working population and findings can therefore not be generalized easily.

Further limitations of this study are a lack of variables which are expected to influence participation behavior such as education or objective work characteristics and the self-selection of participants which is likely to lead to an overestimation of participation rate.

However, with regard to findings from binary and multivariate analysis, several substantial factors influencing participation in health measures could be identified. All health measures are attended more by civil servants than by soldiers. This might be partially affected by different gender ratios in those occupational status groups. Women - who showed an overall higher participation rate - account for about 60% of the civil staff whereas 90% of the military staff is male. However, soldiers also have the option to engage in physical activity during working hours and might thus be less interested in additional offers. This hypothesis is substantiated by the fact that the largest difference in participation between military and civil staff exists for physical activity measures which account for the majority of measures offered. It cannot be ruled out that the group of military servants would show higher participation rates if there were measures which correspond better their demand. Whereas in almost all departments a wide range of physical activity measures was offered, there was little choice of the other measures. A larger selection would satisfy their requirements and result in higher participation rates. Still, the overall participation rate is comparatively high for health measures.

The finding that the health status has no substantial effect on participation seems counterintuitive, but is in line with previous findings. With regard to this result it should be also examined whether the supply of health measures does not meet the needs of this group or if a perceived worse health status is a result of a less health-oriented behavior which would also account for lower interest in participation at health measures.

The hypothesis that employees who evaluate their working characteristics more positive are also more likely to participate in health measures could partially be verified. The rating scale “work demand” turned out to be a decisive factor even when further individual as well as context variables were included in the model. The findings lead to the assumption that higher satisfaction with working characteristics engenders participation at health measures.

Regarding a considerable between-context-variance it can further be assumed that those effects vary between settings. Though the amount of unexplained between-context-variance was reduced when adding further explanatory variables, there are still differences in participation on department level that cannot be explained. Only the number of employees proved to explain participation behavior. As expected, employees in departments with fewer employees are more likely to participate. The proportion of women or military servants did not explain individuals´ participation. Therefore, it seems like not the employee structure but the number of employees determines participation at health measures, which is in line with previous findings [15, 21]^.^ One explanation might be higher social pressure in small units. Furthermore, it can be expected that there is a better information flow in small units because of short and direct communication.

A correlation between context variables and individuals´ assessment of work characteristics was not found. Though, we cannot preclude that future studies which control for further variables on both levels (e.g. working hour policy, awareness of WHP) would not find cross level effects. It would be rather surprising if individual perception was independent from workplace conditions.

## Conclusion

The aim of this study was to examine the influence of working characteristics on the participation in health measures. It could be shown that employees who rated the demands of their work as stressful were also less likely to take part in a health measure. It can therefore be presumed that an improvement of working characteristics aiming at a reduction of the work demand could enhance the willingness to participate. Moreover, the results indicate that characteristics vary between contexts and that the belonging to a certain department influences participation as well. The number of employees seems to be a decisive factor. However, regarding a considerable proportion of between-context variance further research to examine how settings determine individual health behavior is necessary.

In the course of WHP participation it appears worthwhile to pay greater attention to factors of conditional prevention as we found higher participation for more satisfied employees.

## Data Availability

The datasets generated and/or analysed during the current study are not publicly available due their confidentiality level but are available from the corresponding author on reasonable request.

## Footnotes

1 For more details of the study design see Sammito et al. (2015) [23].

2 As measure of internal consistency we estimated Cronbach’s Alpha (α) which is calculated as the mean item inter-correlation [24].

## References

[1] World Health Organization. Ottawa Charta for health promotion. Ottawa: WHO; 1986. http://www.euro.who.int/__data/assets/pdf_file/0004/129532/Ottawa_Charter.pdf. Accessed 31 Jan 2017.

[2] World Health Organization. Health Promotion Glossary. Geneva. http://www.who.int/healthpromotion/about/HPR%20Glossary%201998.pdf. Published 1998. Accessed 20 Jan 2017.

[3] Engelmann F, Halkow A. Der Setting-Ansatz in der Gesundheitsförderung: Genealogie, Konzeption, Praxis, Evidenzbasierung. WZB Discussion Paper. No. SP I 2008-302. http://hdl.handle.net/10419/47403. Accessed 11 Nov 2016.

[4] Wynd CA, Ryan-Wenger NA (2004). Factors predicting health behaviors among Army reserve, active duty Army, and civilian hospital employees. Mil Med.

[5] Sallis JF, Bauman A, Pratt M (1998). Environmental and policy interventions to promote physical activity. Am J Prev Med.

[6] Dooris M (2009). Holistic and sustainable health improvement: the contribution of the settings-based approach to health promotion. Perspectives in Public Health.

[7] Bundeszentrale für gesundheitliche Aufklärung. Fehr R. Ökologische und humanökologische Perspektive. http://www.leitbegriffe.bzga.de/systematisches-verzeichnis/strategien-handlungsansaetze-und-methoden/oekologische-und-humanoekologische-perspektive/. Accessed 28 Nov 2016.

[8] Engbers LH, van Poppel MNM, Chin A, Paw MJM, van Mechelen W (2005). Worksite health promotion programs with environmental changes: a systematic review. Am J Prev Med.

[9] Robroek SJW, van Lenthe FJ, van Empelen P, Burdorf A (2009). Determinants of participation in worksite health promotion programmes: a systematic review. Int J Behav Nutr Phys Act.

[10] Sorensen G, Stoddard A, Ockene JK, Hunt MK, Youngstrom R (1996). Worker participation in an integrated health promotion/health protection program: results from the WellWorks project. Health Educ Behav.

[11] Lewis RJ, Huebner WW, Yarborough CM (1996). Characteristics of participants and nonparticipants in worksite health promotion. Am J Health Promot.

[12] Lerman Y, Shemer J (1996). Epidemiologic characteristics of participants and nonparticipants in health-promotion programs. J Occup Environ Med.

[13] Lassen A, Bruselius-Jensen M, Sommer HM, Thorsen AV, Trolle E (2007). Factors influencing participation rates and employees' attitudes toward promoting healthy eating at blue-collar worksites. Health Educ Res.

[14] Wittig P, Nöllenheidt C, Brenscheidt S; Bundesanstalt für Arbeitsschutz und Arbeitsmedizin. Grundauswertung der BIBB/BAuA-Erwerbstätigenbefragung 2012 - Männer/Frauen in Vollzeit mit den Schwerpunkten Arbeitsbedingungen, Arbeitsbelastungen und gesundheitliche Beschwerden . http://www.baua.de/de/Publikationen/Fachbeitraege/Gd73.html Published 2013. Accessed 6 Dec 2016.

[15] Köper B, Siefer A, Beerman B, Bandura B, Schröder H, Klose J, Macco K (2010). Geschlechtsspezifische Differenzierung von BGF-Konzepten. Fehlzeiten-Report 2010 Vielfalt managen: Gesundheit fördern – Potenziale nutzen.

[16] Nader M. Bindung und Dropout in betrieblichen Gesundheitsförderungsprogrammen. Eine empirische Untersuchung aus der Perspektive der Dienstnehmer/innen am Beispiel des Programms „Aktiv gesund im Betreib” der ASKÖ, Masterarbeit. Wien, Universität: http://othes.univie.ac.at/29847/. Published 2013, Accessed 11 Nov 2016.

[17] Cocker KA, Bourdeaudhuij IM, Cardon GM (2010). The effect of a multi-strategy workplace physical activity intervention promoting pedometer use and step count increase. Health Educ Res.

[18] Nöhammer E, Schusterschitz C, Stummer H (2009). Nutzenpotenziale und Effekte betrieblicher Gesundheitsförderung. Gruppendyn Organisationsberat.

[19] Rojatz D, Merchant A, Nitsch M (2015). Zentrale Einflussfaktoren der betrieblichen Gesundheitsförderung: Ein systematischer Literaturreview. Präv Gesundheitsf.

[20] Robroek Suzan JW, Lindeboom Dennis EM, Burdorf Alex (2012). Initial and Sustained Participation in an Internet-delivered Long-term Worksite Health Promotion Program on Physical Activity and Nutrition. Journal of Medical Internet Research.

[21] Beck D, Lenhardt U (2014). Betriebliche Gesundheitsförderung in Deutschland: Verbreitung und Inanspruchnahme. Ergebnisse der BIBB/BAuA-Erwerbstätigenbefragungen 2006 und 2012. Gesundheitswesen..

[22] van Berkel J, Boot CRL, Proper KI, Bongers PM, van der Beek AJ (2013). Process evaluation of a workplace health promotion intervention aimed at improving work engagement and energy balance. J Occup Environ Med.

[23] Sammito S, Schlattmann A, Felfe J (2015). Betriebliches Gesundheitsmanagement im Geschäftsbereich des Bundesministeriums der Verteidigung – Wissenschaftliche Begleitung eines ehrgeizigen Projektes. Wehrmed..

[24] Kopp J, Lois D (2014). Sozialwissenschaftliche Datenanalyse. Eine Einführung.

[25] Philainen K, Santtila M, Häkkinen K, Lindholm H, Kyröläinen H (2014). Cardiorespiratory responses induced by various military field tasks. Mil Med.

[26] Jonas WB, O'Connor FG, Deuster P (2010). Why Total force fitness?. Mil Med.

[27] Bergmann BP, Mackay DF, Pell JP (2015). Long-term consequences of alcohol misuse in Scottish military veterans. Occup Environ Med.

[28] McDowell MA, van Hubbard S (2013). Adherence to national diet and physical activity objectives among active duty military personnel: what are the implications?. J Acad Nutr Dietetics.

[29] Naghii MR (2006). The importance of body weight Management for Military Personnel. Mil Med.

[30] Plat MJ, Frings-Dresen MHW, Sluiter JK (2011). A systematic review of job-specific workers’ health surveillance activities for fire-fighting, ambulance, police and military personnel. Int Arch Occup Environ Health.

[31] Bahadori Mohammadkarim, Sanaeinasab Hormoz, Ghanei Mostafa, Mehrabi Tavana Ali, Ravangard Ramin, Karamali Mazyar (2015). The Social Determinants of Health in Military Forces of Iran: A Qualitative Study. Journal of Environmental and Public Health.

